# BRAF Mutations in an Italian Regional Population: Implications for the Therapy of Thyroid Cancer

**DOI:** 10.1155/2015/138734

**Published:** 2015-11-26

**Authors:** Eleonora Monti, Michela Bovero, Lorenzo Mortara, Giorgia Pera, Simonetta Zupo, Elena Gugiatti, Mariella Dono, Barbara Massa, Gian Luca Ansaldo, Giusti Massimo

**Affiliations:** ^1^Department of Internal Medicine, Endocrinology Unit, IRCCS IST Azienda Ospedaliera Universitaria “San Martino”, Largo R. Benzi, No. 10, 16132 Genoa, Italy; ^2^Department of Pathology, Molecular Diagnostic Unit, IRCCS IST Azienda Ospedaliera Universitaria “San Martino”, Largo R. Benzi, No. 10, 16132 Genoa, Italy; ^3^Department of Surgery, Endocrinology Surgery Unit, IRCCS IST Azienda Ospedaliera Universitaria “San Martino”, Largo R. Benzi, No. 10, 16132 Genoa, Italy

## Abstract

*Background*. Molecular diagnostics has offered new techniques for searching for mutations in thyroid indeterminate lesions. The study's aim was to evaluate the BRAF mutations' incidence in an Italian regional population.* Subjects and Methods*. 70 Caucasian patients born in Liguria with indeterminate or suspicious cytological diagnoses.* Results.* A BRAF gene mutation was successfully analyzed in 56/70 patients. The mutation was BRAF V600E in 12/56 cases (21%) and BRAF K601E in 2/56 (4%). Of the BRAF mutated samples on cytological diagnosis (14/56 cases), 2/14 cases (14%) were benign on final histology and 12/14 (86%) were malignant. All BRAF-mutated cases on cytology that were found to be benign on histological examination carried the K601E mutation. Of the nonmutated BRAF cases (42/56, 75%) which were later found to be malignant on definitive histology, 5 cases were follicular carcinomas (36%), 3 cases were incidentally found to be papillary microcarcinomas (22%), 2 were cases papillary carcinomas (14%), 1 was case follicular variant of papillary carcinoma (7%), 1 was case medullary carcinoma (7%), 1 case was Hurtle cell tumor (7%), and 1 case was combined cell carcinoma and papillary oncocytic carcinoma (7%).* Conclusions*. The presence of the BRAF V600E mutation may suggest a more aggressive surgical approach. BRAF K601E mutation did not correlate with malignancy indexes.

## 1. Introduction

Thyroid carcinoma is the most common endocrine neoplasm and is increasing worldwide (in the USA, 8.7 per 100,000 people) [1–4]. Papillary thyroid carcinomas (PTCs) accounted for 74% of all thyroid carcinomas in 1973 and 87% in 2003. In this period, its incidence (including that of the follicular variant of PTC) increased by 189%; the rate of follicular carcinomas remained stable, and the rate of anaplastic carcinoma decreased by 22% [2]. Thyroid cancer may be found in thyroid nodules, the nature of which can be investigated by means of cytological examination (fine-needle aspiration biopsy, FNAB). Most thyroid nodules are benign (85%) and in most cases it is possible to distinguish clearly between benign and malignant nodules [5, 6]. On cytological examination, however, there remains a “gray zone” comprising thyroid nodules classified as Thy 3-Thy 4 (according to the classification of the British Thyroid Association), the diagnosis of which is indefinite between benignity and malignancy (25% of Thy 3 and 70% of Thy 4 cases are malignant on final histology) [7–9]. Recently, molecular diagnostics has offered new techniques for detecting the most common mutations in thyroid cancer (BRAF, RAS, RET/PTC, and PAX 8/PPAR *γ*) in order to optimize the management of indeterminate follicular lesions and to guide the therapeutic approach more appropriately [10–13]. The BRAF gene encoding serine/threonine kinase is regulated by RAS, which mediates the pathway of cellular growth and malignant transformation. The most common mutation in PTC is BRAF V600E (substitution of a thymine with an adenine at nucleotide 1799 and, consequently, substitution, on the transcribed protein at residue 600, of a valine with a glutamate), which is detected in 50% of PTCs on definitive histological diagnosis [14, 15]; searching for this mutation is therefore extremely useful. There are other mutations of the BRAF gene, such as K601E, which displays lower oncogenic activity* in vitro* than V600E; indeed, the kinase activity of V600E is 2.5 times greater than that of the K601E that has been identified in follicular adenomas and, more rarely, in some follicular carcinomas [16, 17]. The incidence of PTC seems to vary, as does the presence of BRAF mutations, according to the amount of alimentary iodine in the population under study [18, 19]. The purpose of our study was to investigate the presence of BRAF mutations in our Ligurian population with a view to modifying the therapeutic approach and follow-up, considering the numerous literature data on the worse prognosis of BRAF-mutated PTCs and their loss of iodine uptake [20–22]. It is common opinion in the literature that molecular cytology following fine-needle aspiration biopsy can usefully guide the therapeutic approach in thyroid nodules with indeterminate follicular lesions (BTA Thy 3-Thy 4) [23–26].

## 2. Materials and Methods

### 2.1. Subjects

Between January 2013 and July 2014, 70 Caucasian out-patients living in Liguria were referred to our “Thyroid Clinic” with an indeterminate cytological diagnosis according to the 2009 BTA classification [27] (Table 1). Cytological-histological correlation was available only in 56/70 (80%) patients (13 men and 43 women; age 20–76 years; average age 51 years; 38 Thy 3 samples, and 18 Thy 4 samples). The thyroid fine-needle aspiration biopsy (FNAB) material was fixed with cytofix on slides, one of which was used for molecular diagnostics. Sonography revealed a uninodular goiter (GUN) in 36 subjects and a multinodular goiter (GMN) in 20 subjects; a thyroditic pattern was discerned in 8 subjects. A clinical condition of acromegaly was present in two patients and primary hyperparathyroidism was diagnosed in one GMN and two GUN. All patients were informed of the method used and provided both written and verbal consent.

### 2.2. Purpose

The purpose of the study was to evaluate the effectiveness of a surgical choice based not only on the cytological diagnosis but also on the detection of BRAF mutations, in our Ligurian population.

The following protocol was adopted:

- If a sample was positive for a BRAF mutation, we suggested total thyroidectomy (with lymphadenectomy if the initial cytological diagnosis was Thy 4; without lymphadenectomy if the initial cytological diagnosis was Thy 3).
- If a sample was negative for the presence of mutation, we chose a less aggressive approach: if the initial cytological diagnosis was Thy 4, we suggested total thyroidectomy without lymphadenectomy; if the initial cytological diagnosis was Thy 3 and no nodular disease was observed in the contralateral lobe, we suggested only lobe-isthmectomy; if the initial cytological diagnosis was Thy 3 but there were nodules in the contralateral lobe and/or chronic thyroiditis, we suggested total thyroidectomy without lymphadenectomy.

### 2.3. Protocol

Thyroids were evaluated by ultrasonography (US) using color Doppler equipment (MyLab Five, Esaote Biomedica, Genoa, Italy) equipped with a 7.5 MHz linear probe. Ultrasound-assisted FNAB was performed with the aid of the same machine. In accordance with the current guidelines for ultrasound [26–28], the nodule parameters evaluated were size, composition (solid or mixed), echogenicity, presence or absence of microcalcified spots, vascularization, margin halo, and irregularity of the margin. Blood samples were taken in the morning between 8 and 9 o'clock. Biochemical evaluation included free thyroid hormones, thyrotropin (TSH), anti-thyroperoxidase antibodies (TPOAb) and calcitonin (CT). TPOAb were determined by means of a commercial assay (Radim) with a cut-off of 100 U/L. TSH and free thyroid hormones were evaluated by means of highly sensitive chemiluminescence (Roche Diagnostics). Normal values were 0.3–4.2 U/L for TSH; 2.7–7.0 pmol/L for free T3 (f-T3); and 11.5–21.8 pmol/L for free T4 (f-T4). CT was determined by means of DiaSorin immunochemiluminescence reagents; in our laboratory the normal value of calcitonin is less than 10 pg/mL.

### 2.4. Molecular Biology Analysis

Somatic point mutation in the BRAF V600 gene was determined on cytological material smeared on a slide after the pathologist had verified the adequacy of the sample and had selected areas with the highest number of neoplastic cells. The presence of nonneoplastic cells, that is, normal thyrocytes, stromal cells, and blood-derived leukocytes, was evaluated to determine the ratio of neoplastic/nonneoplastic cellular compartment. Only areas with a neoplastic/nonneoplastic cells ratio of >50% were considered to be suitable for molecular testing. After removal of the coverslip (48–72 hours), DNA was extracted from selected areas by means of a “home-made” buffer (pH 8, 1% tween) and digestion with Proteinase K, as recommended (Qiagen, Hilden, Germany). The optimal number of cells suitable for molecular studies should be 100 or more. Two methods were used to study the mutation: (i) conventional PCR followed by the direct Sanger sequencing method (according to the recommendations of the Italian Association of Medical Oncology (AIOM) and the Italian Society of Pathology and Cytology (SIAPEC)); and (ii) Real Time PCR (RT PCR) with commercial kits approved for clinical use (therascreen BRAF RGQ PCR, Qiagen).

Briefly, 100–200 ng of genomic DNA was amplified by PCR using 1.5 U Platinum Taq DNA polymerase (Thermo Fisher Scientific, TFS, Milan, Italy), 1x buffer, 2 mM MgCl_2_, 200 nM dNTPs, 30 pmoles of forward and reverse primers in a final volume of 50 *μ*L. The set of primers (BRAF 15 forward: 5′ tgcttgctctgataggaaaatg and BRAF 15 reverse: 5′ agcatctcagggccaaaaat) was used to amplify the entire codon region of exon 15 of the BRAF gene and produce amplicons of 230 bp. The amplified PCR products were then treated with ExoSap (GE Healthcare, Waukesha, USA) as recommended and both strands sequenced by means of dye terminator cycle sequencing (BigDye Terminator v3.1, TFS). Nucleotide sequence detection was performed on an ABI Prism 3130 Genetic Analyzer (TFS), according to standard protocols. The sequence data were analyzed by means of Mac Vector software version 11 (MacVector Inc., North Carolina, USA) in order to identify mutations and to assign genotypes to individual DNA samples. A BRAF mutation was assigned only when at least 3/4 sequences from two independent PCR amplifications yielded the same result.

The therascreen BRAF RGQ PCR Kit is a molecular diagnostic tool for detection of the 4 different V600 mutations (V600E, V600D, V600K, and V600R, including the V600E complex) and utilizes two technologies: ARMS (Amplification Refractory Mutation System), which allows mutation-specific amplification, and Scorpions probes for the detection of amplification. This combination of techniques provides high sensitivity and high specificity. The Real Time PCR protocol and analysis of the data were performed on a Rotor-Gene Q MDx 5 plex HRM instrument by using the Rotor-Gene Q software v. 2.2.3 according to the manufacturer's instructions.

The sensitivity of Sanger sequencing was about 12.5% mutated DNA/wt DNA, as determined in home-made experiments described in [29], while the Limit Of Detection (LOD) of the RT PCR commercial kit ranges from 1.82% to 4.85% among the different V600 mutations, as indicated by the manufacturer (therascreen BRAF RGQ PCR kit handbook).

Only PCR assay and Sanger sequencing were able to detect the K601E mutation.

The choice of the method used for detection of BRAF mutations was based on the material and the amount of DNA extracted and assayed. Whenever possible, both procedures were performed. The laboratory was accredited by Bureau Veritas ISO (International Organization for Standardization) 9001: 2008 and the external quality control for the determination of BRAF mutations was promoted by AIOM in 2014 SIAPEC.

### 2.5. Statistical Analysis

Statistical evaluation (Prism 6.0, GraphPad) of the correlations among cytology, histology, and molecular analysis was performed on fully evaluable patients. All data are reported as mean ± standard deviation (SD) unless otherwise specified. Continuous data were compared by means of nonparametric statistical tests. Percentages were compared by means of Fisher's exact test. Correlations between continuous variables were determined by means of Spearman's test. *P* values <0.05 were considered significant.

## 3. Results

The cytological diagnosis was undetermined in 70 patients; however, cytohistological correlation was available for only 57/70 patients (81.4%). In only one case was the material insufficient for mutations to be sought. A higher percentage of BRAF mutations were found in Thy 4 lesions (8/18 cases, 44%) than in Thy 3 lesions (6/38 cases, 16%) (Table 2).

The nucleotide sequence of the entire exon 15 region of the BRAF gene was obtained in all 56 cases, while RT PCR was performed in 32/56 cases (57%). Mutations in the BRAF gene were detected in 14/56 cases (25%): 2/14 (14%) males and 12/14 (86%) females. In 12/14 cases (86%), the mutation identified was BRAF V600E, while in 2/14 cases (14%) BRAF K601E was detected. Notably, there was 100% concordance between the two different methods used for the detection of V600E mutations, that is, Sanger sequencing and Real Time PCR (32/32 cases).

A similar rate of V600E BRAF mutations was found on Sanger sequencing and RT PCR (12/56 mutated cases (21.4%) and 5/32 mutated cases (15.6%), resp.). The relative slightly high percentage of the V600E mutation rate among the cases analyzed by means of the sequencing method can be ascribed to the fact that the cases that proved BRAF nonmutated on sequencing were then preferentially selected for RT PCR. Of those cases in which molecular analysis of cytology specimens revealed a BRAF mutation, 2/14 (14%) proved benign on histology, while 12/14 (86%) were malignant neoplasms: 11/12 (92%) PTC and 1/12 (8%) PTC follicular variant;* no follicular or medullar neoplasms were found*. BRAF-mutated cases that were found to be benign on final histology carried the K601E mutation.

Of the nonmutated BRAF cases (42/56 cases, 75%) which were later found to be malignant on definitive histology (14 cases), 5 were follicular carcinomas (36%), 3 were incidentally found papillary microcarcinomas (22%), 2 were classic papillary carcinomas (14%), 1 was a follicular variant of papillary carcinomas (7%), 1 was a medullary carcinoma (7%), 1 was a Hurtle cell tumor (7%), and 1 as a combined cell carcinoma and papillary oncocytic carcinoma (7%) (Table 2).

If we consider only the classical variant of papillary carcinoma, a correlation between BRAF V600E mutation and a histological diagnosis of malignancy was found in 11/12 (92%) of cases. No false positive results were recorded in our series.

As a result of BRAF gene analysis, the surgical approach was changed in 17/56 patients (30% of cases). These patients had Thy 3 cytology and nonmutated BRAF gene; instead of thyroidectomy, they underwent lobe-isthmectomy alone: the histological diagnosis was benign in all these cases. The decision to undertake more conservative surgery was based on (i) Thy 3 nodule cytology, (ii) wild-type BRAF gene, and (iii) absence of ultrasound signs of malignancy. There was discordance between the endocrinological indication and the type of surgery performed in 2/56 cases (3.5%). This discrepancy involved only patients with Thy 3 nodules on cytology without the presence of multinodular goiter or thyroiditis; in these cases, a more aggressive surgical approach was adopted. The reasons for adopting a more aggressive approach were the following: in 1 case, the presence at ultrasound of a suspect lymph node which subsequently proved positive for metastasis from a papillary carcinoma and, in the other case, the intraoperative suspicion of malignancy (final histology of this case: Hurtle cell tumor).

The mean size of the nodules that underwent FNAB was 20.2 ± 2.2 mm (median 18 mm, range 5–80 mm). The patients involved were categorized by nodule size (no significant differences in terms of number, gender, average age, sonographic features, or thyroid hormone values emerged among these size categories). The percentage of patients on L-T4 treatment was significantly (*P* = 0.01) higher in the Thy 3 cytology group (11/38 cases, 29%) than in the Thy 4 group (0%). The size of the nodule subjected to FNAB and the ultrasound score were similar in the two groups of subjects. The presence of the BRAF V600E mutation correlated with the Thy classification, being positively correlated with a Thy 4 cytological diagnosis (*P* < 0.0001). By contrast, no correlation was seen between the presence of this mutation and any of the following parameters: LT4 hormone therapy, TSH, f-T4 and calcitonin values, the diameter of the nodule examined, age, or sex. The correlation between the ultrasound score of the nodule and the presence of the BRAF mutation was not statistically significant. However, as the numerical value of *P* (0.07) was very close to the limit considered, we can assume that a statistically significant correlation would emerge if a larger group of patients were analyzed. The correlation between BRAF V600E mutation and malignant histology was statistically significant (*P* = 0.0021).

## 4. Discussion

Situated in the northwest of Italy, Liguria is a region with a population of 1,583,628. The region is bounded on the south by the Ligurian Sea, on the west by France (Provence-Alpes-Cote d'Azur), on the north and east by Piedmont and Emilia Romagna, and on the southeast by Tuscany. The region is part of the Mediterranean Alps.

Thyroid nodules with indeterminate cytology have been studied for many years, with the aim of orienting surgery more accurately and reducing the number of total thyroidectomies for benign thyroid nodules (80% of Thy 3 lesions proved benign in our data).

BRAF mutations have been studied in various Italian regions. Research by Guerra et al. evaluated the frequency of the BRAF V600E mutation in thyroid nodules diagnosed in the Naples area and highlighted the importance of such research in that area [28]. In 2014, Pelizzo et al. analyzed 931 histological samples of PTC in northern Italy (Padua and Trieste) and found a correlation between poor prognosis and the presence of this mutation [30]. Rossi et al. studied the presence of the BRAF V600E mutation in Lazio (Rome), where they observed that the increased sensitivity and specificity of sickle-shaped nuclei were predictive of mutation [31]. Again in 2014, Rossi et al. (Ferrara) analyzed 140 indeterminate lesions and concluded that BRAF mutation testing was an important contribution to cancer diagnosis in their region [32].

It should be borne in mind that BRAF gene mutation, besides genetic and racial factors, seems to be influenced by the geographic area, as is testified by the percentage of papillary carcinomas in the population studied [33], and appears to be correlated with the amount of iodine in the diet [34]. No such epidemiological study has ever been conducted in Liguria.

A recent paper by our team [35] also highlighted the importance of ultrasound elastosonography (USE) in nodules with indeterminate cytology. In that study, we enrolled 63 patients with Thy 3 lesions in which cytological-histological correlation was available and demonstrated that evaluating the ELX 2/1 index in conjunction with conventional ultrasonography was able to reduce the number of thyroidectomies. The cumulative presurgical analysis of Thy 3 nodules (by means of US, USE, and contrast-enhanced US) improved specificity.

Several studies [36–39] have shown a strong correlation between the presence of BRAF mutation and lymph node metastasis, extrathyroid extension [40], advanced stage (III and IV) on diagnosis, and disease recurrence. Furthermore, the mutation would appear to be associated with a lower uptake of iodine and refractoriness to RAI in relapsing PTC [41].

In 2009 [10], Nikiforov published one of the papers that prompted the search for mutations on FNAB cytology. His study involved 84 patients with cytologically indeterminate lesions, in 97% of which was a correlation between the presence of mutations on cytology and malignant histology. In the same year, he searched for 32 mutations in 470 samples from FNAB; this study revealed the importance of searching for other mutations, such as RET/PTC, RAS, and PAX8/PPAR *γ*, in addition to BRAF, in order to increase the sensitivity of the method. Nikiforova also found that molecular research reduced false negative samples on thyroid cytology from 2.1% to 0.9% [42].

In a recent paper, Yip et al. proposed a clinical algorithm based not only on the initial cytology but also on mutational research, as a guide to more or less radical surgery. Moreover, Yip recorded a considerably lower frequency of thyroid carcinomas in patients undergoing lobectomy if presurgical mutational research had been carried out [43].

A multicenter study conducted in 2013 confirmed the close correlation between the presence of a BRAF mutation and increased mortality in PTC patients [44].

A recent study by Liu et al. [45] again emphasized the importance of molecular research. On the basis of the results obtained, these authors proposed a different approach, not only to surgery but also to postsurgical treatment, and suggested the possibility of introducing personalized targeted therapy.

Another recent paper demonstrated the possibility of searching for mutations not only on fresh FNAB material but also in air-dried samples [46]. Having retrospectively analyzed 310 consecutive patients who had undergone surgery for thyroid nodules, these authors found that the sensitivity of FNAB increased from 67% to 75% when mutational research was carried out on air-dried samples [46].

The latest guidelines also suggest (recommendation rating: C) the use of molecular markers (e.g., BRAF, RAS, RET/PTC, and PAX8-PPAR) in the management of thyroid nodules with indeterminate cytology [47].

Thus, on the basis of the data reported, it seems that patients with a BRAF V600E mutation on thyroid FNAB cytology should undergo more aggressive surgery, at least if a microcarcinoma is diagnosed [48, 49], followed by more radical postsurgical treatment [36–51].

The data obtained from our population, who had never undergone mutational study, seem to support the suggestion made in the literature, that is, more aggressive initial surgery and closer follow-up. It should also be emphasized that, on considering only the classical variant of papillary carcinoma in our population, we found a correlation between mutations on cytology and a histological diagnosis of malignancy in a high percentage of cases (92%). Another important finding is that no false positive results were recorded in our series.

In conclusion, in agreement with the literature data, the data from our population suggest that the presence of the BRAF V600E mutation should determine a more aggressive surgical approach (currently adopted only in cases of papillary carcinoma on final histology). In our limited number of cases, the BRAF K601E mutation did not correlate with malignancy indexes. A retrospective study to determine whether patients with the BRAF V600E mutation have a worse outcome than those with wild-type BRAF is currently underway.

The limitation of our study is the relatively small number of cases. In a larger population, we might have been able to discern a correlation between the ELX index and both BRAF V600E and RAS mutations.

## Figures and Tables

**Table 1:** Clinical and pathological characteristics.

Patient characteristics	*N*.
Age (years)	51.4 ± 16.2
Sex:	
Male	13/57 (22.8%)
Female	44/57 (77.2%)
US diagnosis:	
GUN	35/57 (61.4%)
GMN	22/57 (38.6%)
Cytology diagnosis:	
Thy 3	39/57 (68.4%)
Thy 4	18/57 (31.6%)

F = female; M = male; GMN = multinodular goiter; GUN = uninodular goiter.

**Table 2:** Correlation of BRAF status and clinicopathological characteristics.

	BRAF status	
	BRAF wild-type	BRAF-mutated
Cytology diagnosis:		
Thy 3	34/38 (90%)	6/38 (16%)
Thy 4	10/18 (56%)	8/18 (44%)
Final histology:		
FTC	5/42 (12%)	0/14 (0%)
PTC	2/42 (5%)	11/14 (79%)
Nodular hyperplasia colloid or follicular adenoma	28/42 (67%)	2/14 (14%)
mPTC	3/42 (7%)	0/14 (0%)
Combined cell carcinoma and PTC	1/42 (2.25%)	0/14 (0%)
MTC	1/42 (2.25%)	0/14 (0%)
Hurtle neoplasia	1/42 (2.25%)	0/14 (0%)
fPTC	1/42 (2.25%)	1/14 (7%)

PTC = papillary thyroid carcinoma; FTC = follicular thyroid carcinoma; MTC = medullary thyroid carcinoma; fPTC = follicular variant of papillary carcinoma; mPTC = papillary microcarcinoma.
